# Intra-Individual Variability of Urinary EGF and Clusterin, and Effect of Frozen Storage on Stability: Results from UVALID

**DOI:** 10.3390/ijms27093838

**Published:** 2026-04-26

**Authors:** Erik Moedt, Rémon Vos, Wenjun Ju, Stephan J. L. Bakker, Marte O. Rygg, Peter Rossing, Michele Provenzano, Lilio Hu, Gaetano La Manna, Jose L. Gorriz, Francesc Moncho-Francés, Tobias B. Huber, Maja Lindenmeyer, Hiddo J. L. Heerspink, Elisabeth Meister

**Affiliations:** 1 Department of Clinical Pharmacy and Pharmacology, University of Groningen, University Medical Center Groningen, 9700RB Groningen, The Netherlands; e.moedt@umcg.nl (E.M.); r.vos@umcg.nl (R.V.); 2 Department of Internal Medicine, University of Michigan, Ann Arbor, MI 48109, USA; wenjunj@med.umich.edu; 3 Department of Internal Medicine, University of Groningen, University Medical Center Groningen, 9700RB Groningen, The Netherlands; s.j.l.bakker@umcg.nl; 4 Steno Diabetes Center Copenhagen, 2730 Herlev, Denmark; marte.opseth.rygg.01@regionh.dk (M.O.R.); peter.rossing@regionh.dk (P.R.); 5 Department of Clinical Medicine, University of Copenhagen, 1165 Copenhagen, Denmark; 6 Department of Medical and Surgical Sciences (DIMEC), Alma Mater Studiorum-University of Bologna, 40126 Bologna, Italy; michele.provenzano@unical.it (M.P.); lilio.hu2@unibo.it (L.H.); gaetano.lamanna@unibo.it (G.L.M.); 7 Nephrology, Dialysis and Renal Transplant Unit, Department of Pharmacy, Health and Nutritional Sciences, University of Calabria, Rende-Hospital ‘SS. Annunziata’, 87100 Cosenza, Italy; 8 Nephrology, Dialysis and Kidney Transplant Unit, IRCCS-Azienda Ospedaliero-Universitaria di Bologna, 40138 Bologna, Italy; 9 Nephrology Department, Hospital Clinico Universitario de Valencia, INCLIVA Biomedical Research Institute, University of Valencia, 46010 Valencia, Spain; jlgorriz@gmail.com (J.L.G.); francescmoncho@gmail.com (F.M.-F.); 10 III. Department of Medicine, University Medical Center Hamburg-Eppendorf, 20251 Hamburg, Germany; tobias.huber@uke.de (T.B.H.); m.lindenmeyer@uke.de (M.L.); e.meister@uke.de (E.M.); 11 Hamburg Center for Kidney Health, University Medical Center Hamburg-Eppendorf, 20251 Hamburg, Germany

**Keywords:** analytical validation, chronic kidney disease, epidermal growth factor, enzyme-linked immunosorbent assay, pre-analytical factors, urinary biomarkers

## Abstract

Urinary epidermal growth factor (uEGF) and clusterin (uCLU) are emerging biomarkers in chronic kidney disease (CKD), but rigorous analytical validation is required before clinical implementation. We evaluated intra-individual variability and long-term storage stability of uEGF and uCLU in CKD. In the prospective, multicenter UVALID study, 60 adults with CKD stages 2–4 underwent urine sampling at three visits over 8 weeks. First-morning and 24-h urine samples were collected to assess intra-individual variability over 24 h, 3 days and 8 weeks. Biomarkers were measured in duplicate by ELISA and normalized to urinary creatinine (/Cr). Inter-laboratory performance was assessed using quality control samples. Stability after 12 and 15 months of storage at −20 °C and −80 °C and the influence of pH were evaluated. Over 24 h, 3 days, and 8 weeks, uEGF/Cr demonstrated low variability and remained stable after long-term storage at both temperatures. In contrast, uCLU/Cr showed greater variability and pronounced instability at −20 °C, whereas stability was preserved at −80 °C. Samples with pH > 6 partially preserved uCLU stability at −20 °C. Inter-laboratory reproducibility was acceptable for uEGF but suboptimal for uCLU at low concentrations. Thus, uEGF showed robust analytical performance, supporting its potential clinical applicability in CKD, whereas uCLU exhibited important analytical and pre-analytical limitations, warranting further assay optimization. These findings underscore the need for rigorous validation to facilitate biomarker implementation in clinical practice.

## 1. Introduction

Chronic kidney disease (CKD) is a growing global health burden, affecting approximately 10% of the adult population and contributing to substantial morbidity, mortality, and healthcare costs [1,2]. Progression of kidney disease and subsequent complications can often be prevented through early detection and timely initiation of effective pharmacotherapy [3]. Biomarkers may play a key role in this regard by supporting early diagnosis, enabling risk stratification, and guiding treatment decisions through pharmacodynamic monitoring [4].

In recent years, novel therapeutic options beyond conventional renin–angiotensin–aldosterone system (RAAS) inhibition, such as sodium–glucose cotransporter-2 inhibitors (SGLT2i) and endothelin receptor antagonists (ERAs), have demonstrated kidney-protective effects in large clinical trials by reducing albuminuria and slowing kidney function decline [5,6,7,8]. However, treatment response varies considerably between individuals, and a substantial proportion of individuals derive limited kidney benefit despite therapy [9]. These therapies are increasingly used in routine clinical care, underscoring the need for pharmacodynamic biomarkers that can monitor treatment response and help tailor therapy to each individual.

Two emerging urinary biomarkers, epidermal growth factor (EGF) and clusterin (CLU), show particular promise in this context. EGF is involved in tubular epithelial regeneration and has been shown to increase during treatment with SGLT2i, such as canagliflozin, with higher urinary EGF (uEGF) levels associated with improved kidney outcomes [10,11]. CLU, a stress-response protein involved in cell survival, is upregulated after tubular injury and shows potential as a marker of ERA treatment response, with reductions in urinary CLU (uCLU) levels correlating with lower kidney damage [12]. However, their broader clinical application remains challenging, as well-defined reference values in healthy populations and the influence of demographic factors are not yet established.

Before clinical implementation, biomarker candidates must undergo rigorous analytical validation, including assessment of intra-individual variability, sample stability under various storage conditions, and the influence of pre-analytical factors [13]. However, many proposed biomarkers lack such rigorous validation, which likely contributes to the limited translation of promising candidates into routine clinical practice [14,15].

In this prospective multicenter UVALID biomarker validation study, we aimed to evaluate the intra-individual variation in urinary EGF and CLU in individuals with CKD over short-term (24 h and 3-day) and longer-term (8-week) periods. We also assessed the stability of biomarker measurements after long-term frozen storage, and potential influences of urine pH on biomarker levels and stability.

## 2. Results

### 2.1. Study Population

We included 60 participants, of whom 19 (32%) had CKD stage 2, 13 (22%) CKD stage 3a, 12 (20%) CKD stage 3b, and 16 (27%) CKD stage 4 (Table 1). Among these, 24 (40%) had fresh urine samples available, which were measured directly and after frozen storage. Baseline characteristics of these 24 participants were similar to those of the full cohort, although median urinary albumin-to-creatinine-ratio (UACR) was lower and the proportion of participants with type 2 diabetes was higher in this subset (Table S1). For the remaining 36 (60.0%) participants, analyses were conducted using only frozen urine samples. Across CKD stages, UACR and uCLU/Cr progressively increased, whereas uEGF/Cr decreased.

### 2.2. Inter-Laboratory Variation

Quality controls (QC) for both uEGF and uCLU were measured at the three centers analyzing urine samples to assess inter-laboratory variation. For uEGF, recoveries across low, medium, and high QCs ranged from 84% to 99%, with a coefficient of variation (CV) between 1.3% and 4.7% (Table 2). For uCLU, recoveries for medium and high QCs ranged from 102% to 120%, with CVs between 2.0% and 8.8%. Low QCs for uCLU had recoveries >120%, with CVs ranging from 3.4% to 12.1%.

### 2.3. Intra-Individual Variation over 24 h, 3 Days, and 8 Weeks

For 12 (50%) participants, daytime and nighttime fresh urine samples were collected to assess 24-h variation. Median concentrations for uEGF/Cr were 6.1 µg/g for both collections, and for uCLU/Cr were 146.7 µg/g and 176.1 µg/g for daytime and nighttime, respectively (Figure S1). For uEGF/Cr, the median absolute difference was −0.1 µg/g (Interquartile range (IQR): −1.0 to 0.2), with a median relative difference of −4.0% (IQR: −21.5 to 2.8). For uCLU/Cr, the median absolute and relative differences were −9.9 µg/g (IQR: −93.6 to 2.6) and −5.9% (IQR: −26.4 to 2.0), respectively.

**Figure 1:**
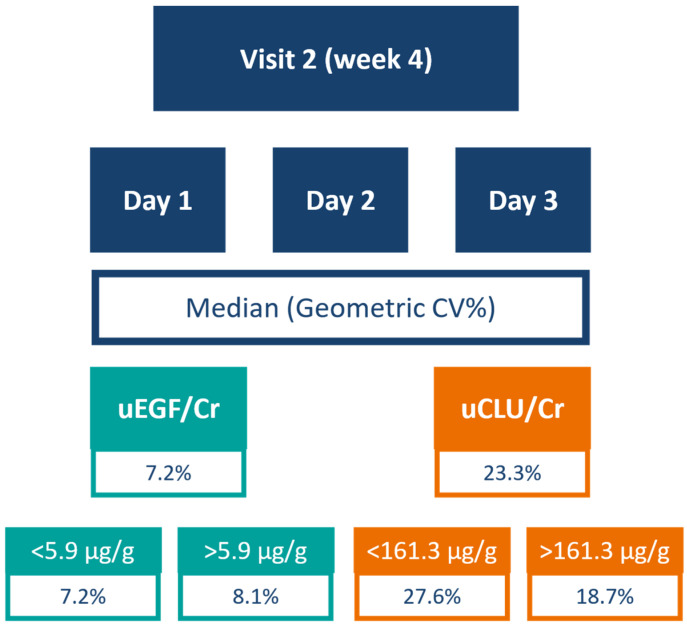
Intra-individual variation over 3 days and median concentrations of uEGF/Cr and uCLU/Cr in FMV samples.

In first-morning void (FMV) urine samples collected over three consecutive days, the median geometric coefficient of variation (GCV) was 7.2% (IQR: 5.6 to 13.7) for uEGF/Cr and 23.3% (IQR: 13.4 to 38.7) for uCLU/Cr (Figure 1). For reference, creatinine showed a median GCV of 18.9% (IQR: 13.8 to 29.6). When stratified by the median uEGF/Cr of 5.9 µg/g at baseline, the median GCV was 7.2% (IQR: 6.2 to 11.5) and 8.1% (IQR: 4.5 to 14.1) for samples below and above the median, respectively. For uCLU/Cr, the median GCV was 27.6% (IQR: 14.7 to 46.6) and 18.7% (IQR: 15.3 to 33.6) below and above the median concentration of 161.3 µg/g, respectively.

**Figure 2:**
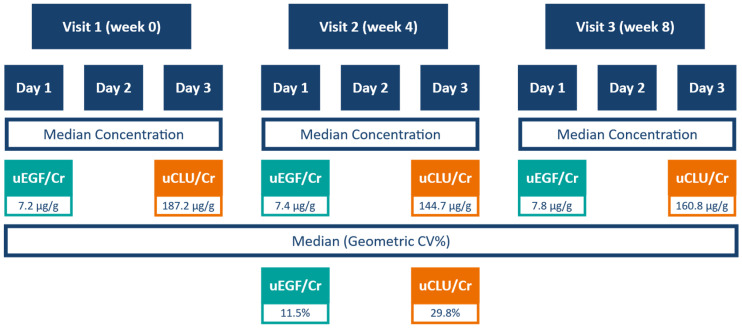
Intra-individual variation over 8 weeks and median concentrations of uEGF/Cr and uCLU/Cr in FMV samples.

Longer-term variation over 8 weeks was assessed using first morning void urine samples collected at visits 1, 2, and 3. Median uEGF/Cr concentrations were 7.2 µg/g, 7.4 µg/g, and 7.8 µg/g, respectively, and median uCLU/Cr concentrations were 187.2 µg/g, 144.7 µg/g, and 160.8 µg/g (Figure 2). The median GCV over 8 weeks was 11.5% (IQR: 7.9 to 17.4) for uEGF/Cr and 29.8% (IQR: 18.9 to 46.5) for uCLU/Cr. For context, creatinine exhibited a median GCV of 20.0% (IQR: 8.2 to 59.9).

### 2.4. Effect of Long-Term Frozen Storage on Stability

**Figure 3:**
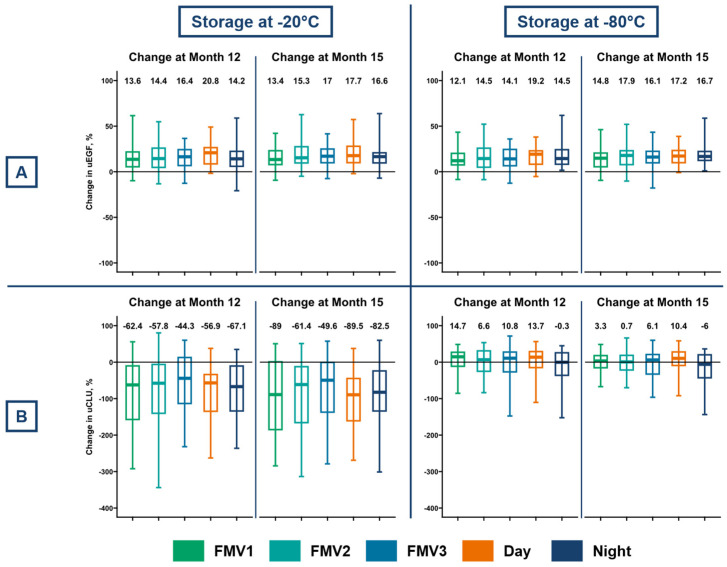
Effect of long-term frozen storage (12 and 15 months) at −20 °C and −80 °C on stability of (**A**) uEGF; and (**B**) uCLU, expressed as median percentage change from baseline. Stability is shown for each aliquot type: first morning urine from days 1 to 3 (FMV1–3), 12-h day, and 12-h night collections. Percentage changes were calculated on the log scale. Boxplots display the median and interquartile range; whiskers indicate 95% confidence intervals.

From baseline to month 12, the median percentage change in uEGF was 14.7% (IQR: 5.7 to 24.2) at −20 °C and 14.1% (IQR: 7.7 to 23.0) at −80 °C (Figure 3A). From baseline to month 15, changes were 16.0% (IQR: 9.3 to 24.9) at −20 °C, and 16.5% (IQR: 7.6 to 22.4) at −80 °C.

For uCLU, the median change from baseline to month 12 was −58.1% (IQR: −141.8 to −4.7) at −20 °C and 11.7% (IQR: −25.6 to 27.6) at −80 °C (Figure 3B). From baseline to month 15, changes were −74.3% (IQR: −164.8 to −10.7) at −20 °C, and 3.6% (IQR: −21.7 to 19.6) at −80 °C. For both uEGF and uCLU, there were no significant differences between aliquots (FMV1, FMV2, FMV3, day, and night).

### 2.5. Subgroup Analyses

Analyses using first morning void urine samples stored at −80 °C showed no major differences in intra-individual variability of uEGF/Cr and uCLU/Cr over 3 days and 8 weeks across subgroups defined by diabetes status, sex, CKD stage, RAASi use, SGLT2i use, or baseline UACR. In contrast, uCLU before normalization to creatinine showed notably higher variability in participants with low UACR, earlier CKD stages, and those without diabetes (Table S2).

### 2.6. Impact of pH

The stability of uEGF did not differ by urine pH at either −20 °C or −80 °C (Figure S2A). For uCLU, storage at −80 °C showed no significant pH-related differences. However, long-term stability at −20 °C was pH-dependent (Figure S2B). At month 12, the median percentage changes were −113.7% (IQR: −222.6 to −20.6) for samples with pH 5, −63.6% (IQR: −77.7 to −12.2) for pH 6, and −13.3% (IQR: −30.0 to 12.5) for pH > 6 (overall *p* = 0.04). At month 15, the corresponding median changes were −122.9% (IQR: −224.3 to −44.9) for pH 5, vs. −55.0% (IQR: −91.7 to −1.3) for pH 6, and −10.9% (IQR: −21.3 to 18.7) for pH > 6 (overall *p* = 0.02).

## 3. Discussion

In this prospective multicenter validation study, we evaluated the analytical performance of uEGF and uCLU in individuals with CKD. Inter-laboratory quality control demonstrated acceptable reproducibility for uEGF, whereas assay performance for uCLU was suboptimal at low concentrations. Intra-individual variability for uEGF/Cr was low over 24 h, 3 days, and 8 weeks, and uEGF remained stable after long-term frozen storage at both −20 °C and −80 °C. In contrast, uCLU/Cr exhibited substantially greater variability, and uCLU showed pronounced instability after storage at −20 °C. Notably, uCLU stability at −20 °C was partially preserved in samples with a urine pH > 6. Patterns of intra-individual variability were consistent across clinically relevant subgroups, supporting the robustness of our findings.

In our study, the analytical profile of uEGF appeared favorable compared with that of albuminuria, the most widely used urinary biomarker in CKD. However, this comparison should be interpreted with caution given the relatively small sample size and use of a single analytical method. Albuminuria is known to exhibit considerable intra-individual variability, with reported CVs in the range of 30–50%, even under standardized conditions [16,17,18]. In contrast, the short-term geometric CV for uEGF/Cr in our study was approximately 7%, indicating substantially lower intra-individual variability. Moreover, uEGF demonstrated good stability after long-term frozen storage at both −20 °C and −80 °C, supporting its feasibility for use in clinical trials and routine care. Together with its strong mechanistic rationale as a marker of tubular regenerative capacity, its consistent association with kidney function decline across multiple cohorts, and its responsiveness to SGLT2 inhibition, these findings support the suitability of uEGF as a pharmacodynamic biomarker for both clinical and research applications [10,11].

Although uCLU remains biologically relevant as a marker of tubular stress and injury, its analytical performance was less favorable and raises concerns. Intra-individual variability of uCLU/Cr was considerably higher than that of uEGF/Cr and appeared comparable to that reported for albuminuria. After storage at −20 °C, uCLU demonstrated reduced stability, particularly at lower concentrations. However, stability was largely preserved when samples were stored at −80 °C, and partial preservation at −20 °C was observed in samples with a urine pH > 6, suggesting that pH modulation may mitigate degradation under certain storage conditions. Similar pH-related effects have been reported for other urinary proteins, including albumin, although results across studies have been inconsistent [19]. These findings are consistent with the suboptimal inter-laboratory quality control performance at low uCLU concentrations, indicating that assay-related factors may contribute to the observed variability. Whether the observed instability reflects intrinsic biochemical properties of uCLU or limitations of the enzyme-linked immunosorbent assay (ELISA) platform used in this study remains uncertain. Further assay optimization and standardization will be required before uCLU can be reliably applied in clinical or translational settings.

Despite a rapidly expanding number of proposed kidney biomarkers, only a small fraction ultimately progress to clinical implementation [20,21,22]. A major barrier is the lack of rigorous analytical validation, with many biomarkers advancing directly from discovery to association studies without adequate assessment of variability, stability, and reproducibility [14,15]. Our study aimed to address this translational gap by systematically evaluating analytical performance characteristics that are prerequisites for clinical adoption. Such implementation-focused validation studies are essential to ensure that biomarkers not only demonstrate biological relevance but also perform reliably under real-world conditions. In this context, biomarker implementation and clinical utility are being investigated within the Personalized Drug Response: Implementation and Evaluation in CKD (PRIME-CKD) consortium [23].

Key strengths of this study include its prospective multicenter design, standardized sampling protocols, evaluation across multiple clinically relevant time scales, and systematic assessment of pre-analytical factors. However, several limitations should be considered. The sample size was modest, and the availability of fresh urine samples varied across participating centers. In addition, analyses relied on commercially available ELISA kits, and results may not be generalizable to alternative assay platforms. Lastly, the absence of well-defined reference ranges for uEGF and uCLU in healthy populations limits the interpretation of absolute biomarker levels.

In conclusion, uEGF demonstrates a favorable analytical profile characterized by low intra-individual variability, acceptable inter-laboratory reproducibility, and stability under commonly used storage conditions, supporting its potential for clinical application in CKD. In contrast, uCLU shows substantial analytical and pre-analytical vulnerabilities that should be addressed before clinical adoption. Overall, our findings underscore the critical importance of rigorous analytical validation as a prerequisite for translating biomarker discoveries into routine clinical practice.

## 4. Materials and Methods

### 4.1. Participants

The UVALID study enrolled 60 adults with established CKD stages 2 to 4. Inclusion criteria were based on GFR categories, aiming for balanced representation across CKD stage 2 (GFR 60–89 mL/min/1.73 m^2^), stage 3a (GFR 45–59 mL/min/1.73 m^2^), stage 3b (GFR 30–44 mL/min/1.73 m^2^), and stage 4 (GFR 15–29 mL/min/1.73 m^2^). Key exclusion criteria included planned kidney donation or dialysis initiation during the study period. Participants were recruited from outpatient clinics and provided informed consent prior to enrollment.

### 4.2. Study Design

UVALID was a prospective, multicenter biomarker validation study conducted at five European sites. Participants were enrolled at four centers in Copenhagen, Bologna, Hamburg, and Valencia, while batch biomarker analyses after frozen storage were performed at a central laboratory at the University Medical Center Groningen. Three study visits were scheduled over an 8-week period, including a baseline visit and two additional visits, each 4 weeks apart. Baseline clinical and demographic data were collected, and vital signs were recorded at each visit. Participants remained clinically stable, with no changes in kidney-related therapy between visits, allowing assessment of intra-individual biomarker variability. Half of the participants collected one 24-h urine sample per visit, divided into two 12-h aliquots, in addition to three consecutive FMV urine samples. FMV samples (FMV1–FMV3), as well as paired daytime and nighttime urine samples, were collected at each visit to assess short-term intra-individual variability and diurnal variation. Two of the four enrolling centers performed immediate analyses of urinary EGF and CLU. Urinary albumin, urinary creatinine, UACR, and dipstick analyses were performed locally on all samples. All samples were aliquoted and stored at −20 °C and −80 °C for subsequent analyses. Ethics approval for the study protocol was obtained by all participating centers, and the trial was conducted in accordance with the Declaration of Helsinki and Good Clinical Practice guidelines.

### 4.3. Biomarker Measurements

Urinary EGF and CLU concentrations were measured using commercial ELISA kits (Human EGF Quantikine ELISA Kit DEG00, R&D Systems, Minneapolis, MN, USA; Human Clusterin ELISA Kit ab174447, Abcam, Cambridge, UK). All measurements were performed in duplicate, and results were averaged. Fresh EGF measurements showed a mean CV of 2.7%, whereas CLU measurements had a mean CV of 4.4%. For frozen samples, EGF measurements had a mean CV of 1.8%, and CLU had a mean CV of 2.7%. Biomarker concentrations were normalized to urinary creatinine (uEGF/Cr and uCLU/Cr) for the assessment of intra-individual variability. Measurements that fell above or below the assay limits of detection or quantification were rare and were repeated using alternative dilutions when encountered. Stability analyses were performed on un-normalized concentrations.

QC samples at low, medium, and high concentrations were included at each analytical center. Recovery percentages and CVs were calculated to assess inter-center reproducibility and analytical precision.

### 4.4. Analytical Performance Measures and Statistical Analysis

Continuous variables with a normal distribution were presented as means and standard deviations (SD). Due to non-parametric distributions, UACR, uEGF, uEGF/Cr, uCLU, and uCLU/Cr were reported as medians and IQR. Categorical variables were shown as frequencies and percentages.

Intra-individual variability was assessed over 24 h, 3 days, and 8 weeks. Diurnal variation was evaluated by comparing biomarker concentrations in paired daytime and nighttime 12-h samples collected at baseline, with median absolute and relative differences reported. Short-term variability over three days was calculated using the GCV across FMV1–3 samples collected at baseline. Longer-term variability over eight weeks was assessed by first calculating the geometric mean over 3 days (FMV1–3) at each of the three visits and then calculating the GCV across these geometric means. This analysis was restricted to participants with complete data at all three visits. For all variability analyses, median (25th to 75th percentile) GCV values are reported because of the skewed distribution.

The effect of long-term frozen storage at −20 °C and −80 °C after 12 and 15 months was evaluated by calculating median percentage changes in EGF and CLU concentrations from baseline. Percentage changes were calculated on the log scale and are reported as log-ratio multiplied by 100. Stability analyses were performed across different sample types (FMV1–3, daytime, nighttime).

The influence of urine pH on biomarker stability was explored by grouping samples into pH categories (5, 6, and >6) and analyzing its association with percentage changes over time using one-way ANOVA.

Results with *p* < 0.05 were considered to be statistically significant. All statistical analyses were conducted with R version 4.4.2. (R project for Statistical Computing, http://www.r-project.org).

## Figures and Tables

**Table 1:** Baseline characteristics of the total population and subgroups defined by CKD stage.

	Total N = 60	CKD 2 (eGFR 89–60) N = 19	CKD 3a (eGFR 59–45) N = 13	CKD 3b (eGFR 44–30) N = 12	CKD 4 (eGFR 29–15) N = 16
Age, years	65.8 (13.2)	61.1 (15.0)	65.1 (12.8)	70.2 (6.40)	68.6 (14.2)
Gender: Male, n (%)	35 (58.3%)	8 (42.1%)	9 (69.2%)	8 (66.7%)	10 (62.5%)
Diabetes: Yes, n (%)	30 (50.0%)	8 (42.1%)	5 (38.5%)	8 (66.7%)	9 (56.2%)
Systolic BP, mmHg	131 (15.3)	127 (12.8)	135 (12.7)	137 (19.1)	126 (15.6)
Hematuria: Yes, n (%)	17 (28.3%)	7 (36.8%)	4 (30.8%)	3 (25.0%)	3 (18.8%)
UACR, mg/g	23.3 [5.83; 178]	8.04 [2.91; 124]	23.7 [5.91; 70.2]	22.4 [5.44; 99.1]	137 [20.8; 347]
ACE-I: Yes, n (%)	20 (33.3%)	5 (26.3%)	3 (23.1%)	5 (41.7%)	7 (43.8%)
ARB: Yes, n (%)	19 (31.7%)	6 (31.6%)	6 (46.2%)	4 (33.3%)	3 (18.8%)
SGLT2: Yes, n (%)	26 (43.3%)	5 (26.3%)	6 (46.2%)	8 (66.7%)	7 (43.8%)

**Table 2:** Comparison of quality control results by analytical center.

	QC High		QC Medium		QC Low	
	Recovery	CV	Recovery	CV	Recovery	CV
**uEGF:**						
Copenhagen	96%	1.3%	98%	2.8%	99%	4.6%
Hamburg	86%	2.9%	84%	3.2%	89%	2.2%
Groningen	93%	1.7%	93%	4.7%	94%	1.4%
**uCLU:**						
Copenhagen	109%	5.7%	120%	8.8%	139%	12.1%
Hamburg	105%	7.9%	108%	6.4%	123%	3.4%
Groningen	102%	2.3%	106%	2.0%	129%	11.8%

## Data Availability

The data presented in this study are available from the corresponding author, H.J.L.H., upon reasonable request, in accordance with ethical and privacy restrictions.

## Supplementary Materials

The following supporting information can be downloaded at: https://www.mdpi.com/article/10.3390/ijms27093838/s1.

## Abbreviations

The following abbreviations are used in this manuscript:

CKD	Chronic kidney disease
RAAS	Renin–angiotensin–aldosterone system
SGLT2i	Sodium–glucose cotransporter-2 inhibitors
ERA	Endothelin receptor antagonists
EGF	Epidermal growth factor
CLU	Clusterin
uEGF	Urinary EGF
uCLU	Urinary CLU
UACR	Urinary albumin-to-creatinine-ratio
QC	Quality control
CV	Coefficient of variation
IQR	Interquartile range
FMV	First-morning void
GCV	Geometric coefficient of variation
ELISA	Enzyme-linked immunosorbent assay
SD	Standard deviation
