# Supplementary material for: Intra-Individual Variability of Urinary EGF and Clusterin, and Effect of Frozen Storage on Stability: Results from UVALID
Source: Int J Mol Sci. 2026 Apr 26;27(9):3838. doi: 10.3390/ijms27093838 (PMC13163508; doi:10.3390/ijms27093838)
Supplement: Supplementary file 1 [file ijms-27-03838-s001.zip › ijms-4250383-supplementary.pdf]

Supplementary Material

Table S1: Baseline characteristics of the total population with fresh urine samples available and subgroups defined by CKD stage 2

Figure S1: Diurnal variation in uEGF/Cr and uCLU/Cr concentrations, shown as median absolute and relative differences. 3

Table S2: Intra-individual variability of uEGF, uEGF/Cr, uCLU and uCLU/Cr over 3 days and 8 weeks across subgroups in samples stored at -80 °C for 12 months 4

Figure S2: Effect of long-term frozen storage (12 and 15 months) at -20 °C and -80 °C on stability of A) uEGF and B) uCLU, stratified by urine pH and expressed as median percentage change from baseline...5

Table S1: Baseline characteristics of the total population with fresh urine samples available and subgroups defined by CKD stage

	Total<br>N=24	CKD 2<br>(eGFR 89-60)<br>N=6	CKD3a<br>(eGFR 59-45)<br>N=6	CKD 3b<br>(eGFR 44-30)<br>N=5	CKD 4<br>(eGFR 29-15)<br>N=7
Age, years	64.3 (13.6)	66.3 (13.4)	62.0 (13.9)	69.2 (5.17)	61.0 (18.3)
Gender: Male, n (%)	15 (62.5%)	4 (66.7%)	4 (66.7%)	3 (60.0%)	4 (57.1%)
Diabetes: Yes, n (%)	17 (70.8%)	5 (83.3%)	2 (33.3%)	5 (100%)	5 (71.4%)
Systolic BP, mmHg	134 (17.3)	133 (10.4)	137 (15.0)	148 (9.67)	121 (21.1)
UACR, mg/g	11.9 [3.42;59.8]	4.93 [2.80;7.75]	13.7 [3.67;44.4]	19.8 [3.56;687]	56.3 [26.4;214]
uEGF FMVs, ng/mL	3.87 [2.52;7.01]	5.69 [4.00;11.9]	7.02 [6.74;7.64]	3.78 [1.97;3.81]	2.17 [0.71;3.35]
uEGF/Cr FMVs, µg/g	5.93 [3.19;7.78]	7.77 [6.45;10.4]	7.84 [7.49;9.49]	4.23 [3.64;4.24]	2.37 [1.96;3.18]
uCLU FMVs, ng/mL	131 [85.1;196]	140 [103;172]	128 [87.1;238]	155 [131;480]	85.1 [47.8;181]
uCLU/Cr FMVs, µg/g	161 [128;213]	144 [137;170]	178 [127;222]	173 [142;1030]	142 [85.1;223]
ACE-I: Yes, n (%)	9 (37.5%)	1 (16.7%)	1 (16.7%)	3 (60.0%)	4 (57.1%)
ARB: Yes, n (%)	9 (37.5%)	3 (50.0%)	3 (50.0%)	1 (20.0%)	2 (28.6%)
SGLT2: Yes, n (%)	14 (58.3%)	3 (50.0%)	3 (50.0%)	4 (80.0%)	4 (57.1%)

Figure S1: Diurnal variation in uEGF/Cr and uCLU/Cr concentrations, shown as median absolute and relative differences.

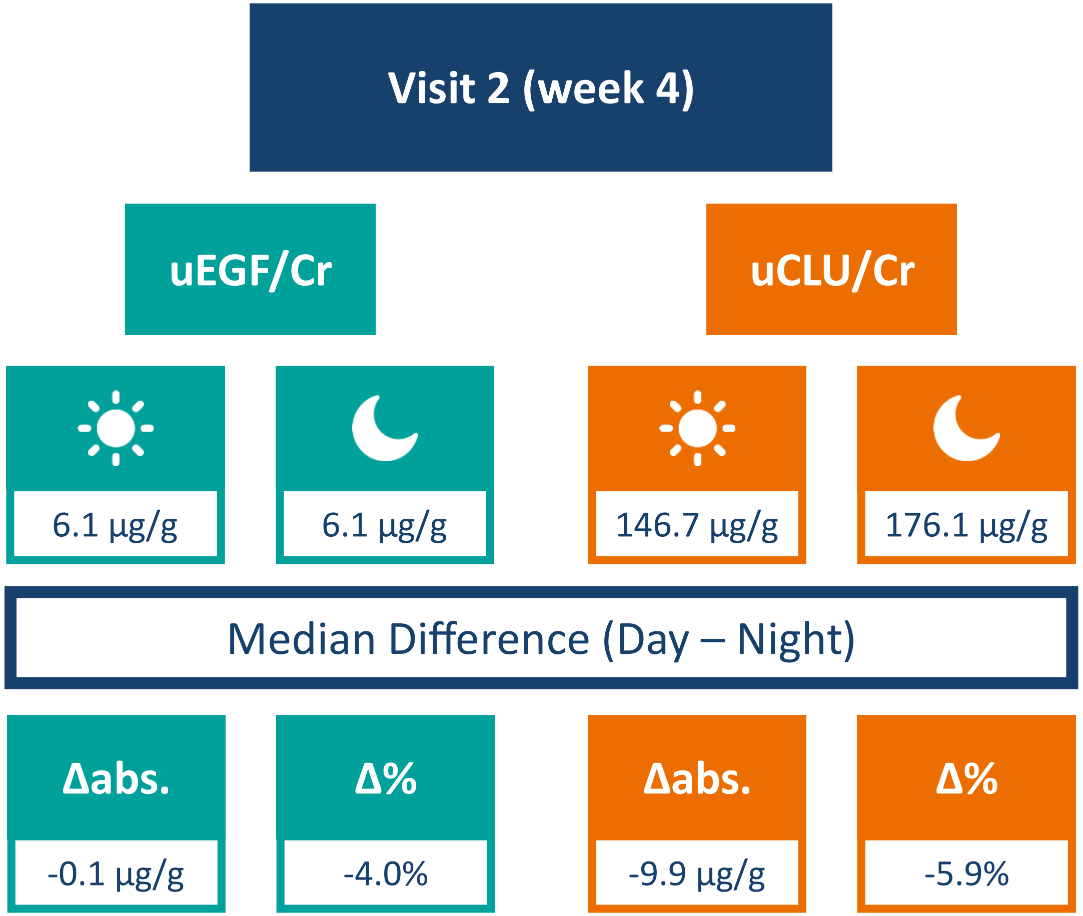

Table S2: Intra-individual variability of uEGF, uEGF/Cr, uCLU and uCLU/Cr over 3 days and 8 weeks across subgroups in samples stored at -80 °C for 12 months

	Variability over 3 days, GCV%					Variability over 8 weeks, GCV%				
**Subgroup**	N	uEGF	uEGF/Cr	uCLU	uCLU/Cr	N	uEGF	uEGF/Cr	uCLU	uCLU/Cr
Overall	58	25.8	8.6	48.9	34	56	21.0	11.7	38.5	31.3
Diabetes: Yes	30	21.1	8.4	32.7	33.2	30	22.3	11.0	33.3	26.5
Diabetes: No	28	29.2	9.6	60.2	35.4	26	20.0	14.8	68.1	50.2
Gender: Male	35	26.3	15.1	42.5	34.2	33	20.2	15.4	34.9	26.6
Gender: Female	23	23.7	7.8	56.5	33.8	23	21.8	10.4	41.7	31.4
CKD Stage: 2	19	30.2	11.2	62.6	43.2	19	26.3	16.5	48.5	35.9
CKD Stage: 3a	12	31.4	8.8	57.6	37.3	11	28.0	11.6	87.4	64.3
CKD Stage: 3b	11	33.5	8.4	42.5	34.6	11	17.8	10.2	34.9	25.1
CKD Stage: 4	16	17.8	6.4	28.4	18.8	15	16.9	11.4	29.4	21.6
RAASi: Yes	38	23.6	8.4	51.8	35.5	37	25.6	11.6	38.2	31.4
RAASi: No	20	36.3	9.9	36.7	32.8	19	18.6	11.8	39.7	31.2
SGLT2i: Yes	26	24.2	8.3	44.4	34.4	26	22.3	10.0	32.2	26.4
SGLT2i: No	32	26.2	9.9	54.3	33.7	30	20.0	16.0	56.8	39.6
UACR: ≤30 mg/g	29	27.8	7.8	61.3	37.8	29	29.3	15.4	59.7	32.9
UACR: >30 mg/g	29	23.2	11.5	32.7	19.6	27	16.1	10.5	32.8	26.6

Figure S2: Effect of long-term frozen storage (12 and 15 months) at -20 °C and -80 °C on stability of A) uEGF and B) uCLU, stratified by urine pH and expressed as median percentage change from baseline

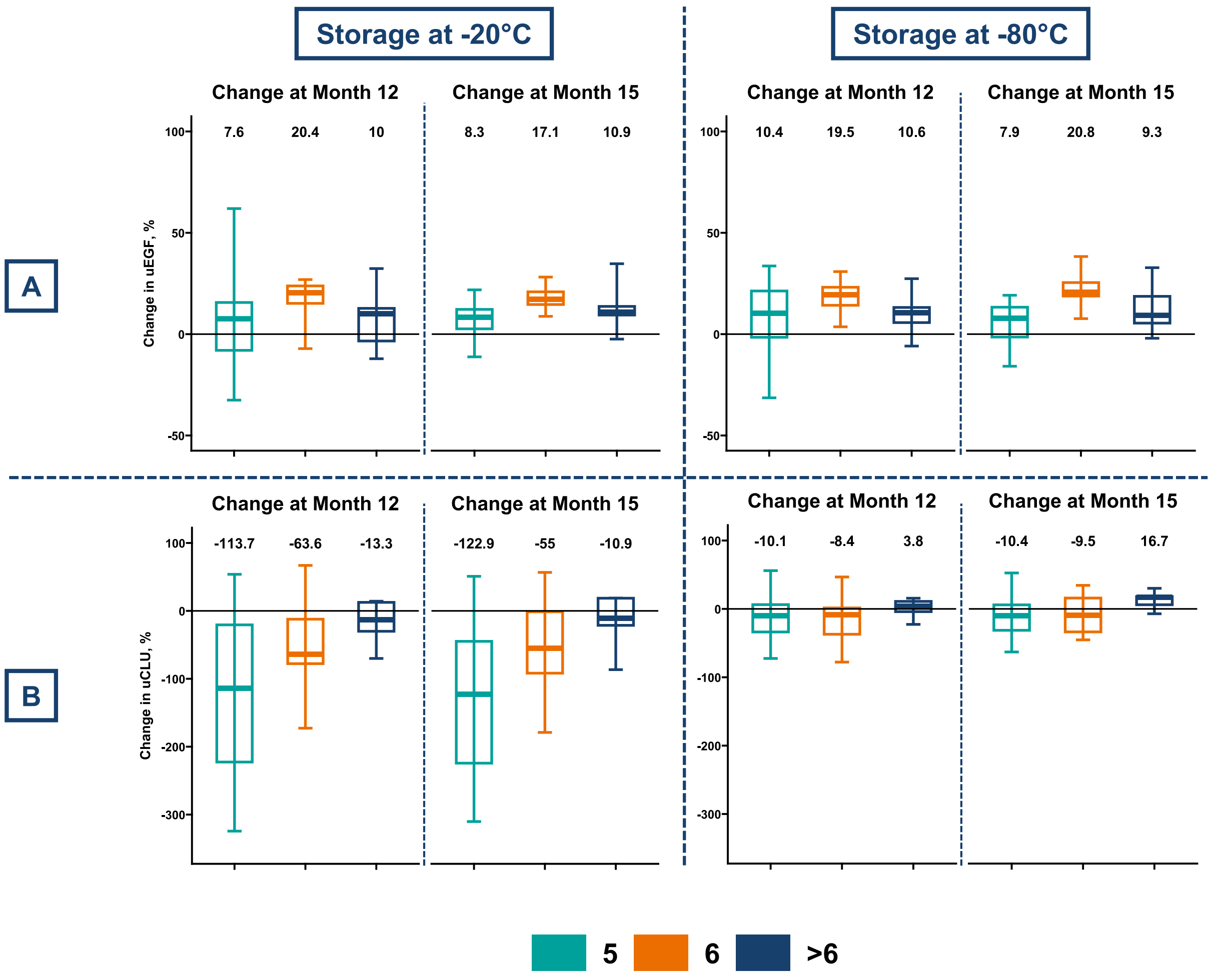

Percentage changes were calculated on the log scale. Boxplots display the median and interquartile range; whiskers indicate 95% confidence intervals
